# Lanoline*Read before the Pennsylvania State Veterinary Medical Association.

**Published:** 1887-04

**Authors:** Alexander Glass


### Art. XVI.—LANOLINE.*

BY DR. ALEXANDER GLASS.

About the beginning of the year 1886, a German physician by the name of Prof. Oscar Liebreich read a very able and exhaustive paper on a new preparation obtained from wool fat called Lanoline; this is a neutral fat of the cholesterine order. Since making known the results of his researches, a number of practical experiments have been made by noted dermatolgists on man, and by myself on a number of animals, and in all cases with excellent results.

It is not exactly new in medicine in one sense, for it was used and spoken of by the ancients under the names of Oesipus who also gave the method of preparation. It was a crude material, and from the method of its preparation very impure, it therefore fell into disuse. The method of preparation is a simple one.

It is an ointment of a light yellow color, or under the name of Aguine a dark preparation is to be had; the former has a slight woolly odor like the sheep, or new woolen cloth, not at all unpleasant, the latter is dark and has a very decided odor. It is pasty and tenacious in character, resembling very thick honey, little or no taste; on exposure to air it turns a bright orange color, probably due to the absortion of the water it contains. The property it posseses of taking up water is surprising, at least 75%, forming a yellow, easily worked ointment; it is said to take up equal parts by weight, but as we obtain it, it generally contains 20 to 30%. Its melting point is about the temperature of the skin, or about 90 to 95°F., which is an excellent property especially for ointments. It is neutral in reaction and it is almost impossible to decompose it; it combines freely with water, oils, fats and glycerine.

As a base it is in several ways far superior to either lard or vaseline, or as it is sometimes called cosmoline or petrolatum. Lard is not neutral, it is readily decomposed even if benzoated and by the formation of fatty acids, oleic and butyric, becomes rancid, and an irritant effect is produced on the affected parts

*Read before the Pennsylvania State Veterinary Medical Association.

to which it is applied; vaseline is neutral and not readily decomposed, but as all paraffine products are very insoluble, the drugs mixed with them are only held mechanically in their bodies and only small parts comes in direct contact with the skin, and is absorbed. Dr. Shoemaker says that the power of vaseline to penetrate is so feeble that it is a question whether it can justly be said to possess such a property.

With Lanoline, on the contrary, the great property is the readiness with which it is taken up and absorbed by the skin, penetrating deeply into the tissues, holding the drug incorporated with it in suspension, carrying it with it and producing an effect on the deeper tissue of the derm, an effect hitherto only obtainable with the oleates or lard, and that not at all perfectly. Lanoline can be used as a base for almost all diseases of the skin, met with in veterinary practice. In acute eczema, combine with boric acid or iodoform, boric acid 10 parts to 90 of Lanoline; and in some cases where a large surface is to be covered, 20 parts of lard may be incorporated with it rendering it soft and more readily applied; or iodoform 10 parts, lard 10 and Lanoline q s 100.

In chronic eczema, frequently seen in the dog, preparations of tar, or the sulphides. Pices Liquida (tar liquid) 15 parts to 85 of Lanoline, or oil of cade 20 parts to 10 of lard and 70 of Lanoline.

In Erythematic diseases as chafes, galls, scratches, an ointment composed of Plumbi acetas, Sulphur, Salicine, each 10 parts, 20 of lard and 50 parts of Lanoline is an excellent preparation. In those extremely painful attacks of mud fever seen in the last month, 5 grains of opium lessens the pain very much. Uticaria, nettle rash, hives, etc., causing often extreme itchiness, 40 parts of simple lead plaster, 10 of lard and 50 of Lanoline act very nicely lessening the pain and drying the small vesicles; for exuberant granulations (proud flesh) 10 of iodoform and 90 of Lanoline.

In the parasitic diseases of animals, (Dermatodectes, Sarcoptes, Demadere folliculorum, etc.,) it is evident that Lanoline fro m its power of deep penetration will carry any destructive drug to such parasite into its home and destroy it; one of the best preparations in such cases is beta-napthoil or in the more crude form some of the tar prepvrations; Oleum pices, oil of Cade, Pices liquida. The preparation of beta-napthoil is 3 parts, 12 of lard, 85 of Lanoline; the animal to be washed thoroughly to remove scabs, etc., and then a small quantity well rubbed into the part, taking care to allow no excess to remain, lest the animal should lick it.

